# Role of free volume in governing electrical transport in V_2_O_5_–Cu_2_O–P_2_O_5_ glass ceramic nanocomposites

**DOI:** 10.1038/s41598-026-65220-1

**Published:** 2026-08-12

**Authors:** D. E. El Refaay, M. M. El-Desoky, Amany E. Harby, Hamdy F. M. Mohamed, E. E. Abdel-Hady, Yehya S. Elsharkawy

**Affiliations:** 1 Department of Physics, Faculty of Science, Suez University, P.O. Box 43221, Suez, Egypt; 2 Physics Department, Faculty of Science, Minia University, P.O. Box 61519, Minia, Egypt

**Keywords:** Materials science, Nanoscience and technology, Physics

## Abstract

Glass–ceramic nanocomposites of xV₂O₅–(40−x)Cu₂O–60P₂O₅ (x = 10–40 mol%) were prepared by melt quenching followed by heat treatment at 873 K. XRD analysis confirmed the formation of Cu₂O and V₂O₅ nanocrystalline phases with crystallite sizes in the range of 16–27 nm embedded within a partially amorphous matrix. DC electrical conductivity increased by nearly four orders of magnitude with increasing V₂O₅ concentration, accompanied by a systematic decrease in activation energy, indicating enhanced thermally activated small-polaron hopping conduction. Positron annihilation lifetime (PAL) spectroscopy revealed a gradual reduction in vacancy-type defect size and free-volume parameters with increasing V₂O₅ content, suggesting improved local structural ordering and reduced carrier localization. In contrast, the oxygen molar volume increased slightly with composition, reflecting macroscopic structural rearrangement and modification of the phosphate network. These observations indicate that the macroscopic network expansion and microscopic reduction of localized free-volume cavities occur simultaneously at different structural scales. The enhancement in electrical conductivity is primarily governed by increased vanadium ion concentration and reduced hopping distance, while defect reduction contributes indirectly through improved structural compactness and orbital overlap between neighboring vanadium ions. The present results provide insight into the correlation between structural evolution, free-volume defects, and electrical transport in transition-metal oxide glass–ceramic nanocomposites.

## Introduction

Glass–ceramic nanocomposites (GCNs) have attracted increasing interest due to the flexibility of glasses and the functionality of the nanocrystals^1–3^. The incorporation of nanocrystals into the glass matrix has been reported to have a profound effect on the electrical conductivity, thermal stability, and optical properties^1,2,4,5^. Vanadium phosphate glasses have been considered as a potential transition metal oxide system, as V₂O₅ not only acts as a glass former and redox center but also facilitates the development of semiconducting properties through the generation of polaron mechanisms^6–8^.

The addition of Cu₂O further modifies the phosphate network by altering connectivity and promoting the formation of non-bridging oxygen (NBO) sites, which influence free-volume distribution and defect structure^9,10^. Transition-metal-oxide (TMO)–containing glasses are well known for their semiconducting properties, where dc electrical conduction is commonly explained by thermally activated electron hopping between different valence states of transition-metal ions^6,11,12^. In vanadate-based glasses, strong electron–lattice interactions favor the formation and localization of small polarons, which govern charge-transport behavior^6,7,11^. Despite these advantages, the relationship between structural modification, defect states, and electrical transport in glass–ceramic nanocomposites remain insufficiently understood. In particular, the role of defects of the vacancy type and free-volume voids in determining electrical conductivity has not been systematically explored. These aspects are important in order to control the charge transport and optimize the performance of solid-state semiconducting and ionic materials.

One of the very sensitive techniques for the characterization of vacancy-type defects and free-volume properties of condensed matter systems is the Positron Annihilation Lifetime (PAL) Spectroscopy^13,14^. Instead of the identification of larger impurity atoms, this technique has been found to be more sensitive for the characterization of vacancies, vacancy clusters, interstitial loops, and grain boundaries due to the preference of protons for areas of lower electron density^13–16^. The correlations of density, free volume, and defects have been established with the successful application of this technique for molecular materials and various glasses as reported in the literature^15–18^. For example, Alharbi et al.^18^ reported the decrease of the o-Ps hole volume with increasing TiO_2_ concentration in glass–ceramic systems, while an inverse relationship between density and vacancy volume fraction has been reported by Saddeek et al.^17^. Only a few studies have reported the correlations of PAL with the electrical and structural properties of phosphate-based GCNs^17–19^.

The existing glass composition possesses a phosphate network with 60 mol% P₂O₅, which is already proven and established as a glass-forming composition that is able to incorporate high concentrations of transition metal oxides^8–10^. The phosphate glass composition possesses a unique ability to form homogeneous glasses even with relatively high concentrations of dopants, mainly because of its high compositional flexibility and its high solubility limits of modifiers such as V₂O₅ and Cu₂O, as per the literature^8–10^. To investigate the systematic changes of progressive substitutions of V₂O₅ for Cu₂O, the composition of interest is deliberately chosen such that it is in the composition range of xV₂O₅–(40−x)Cu₂O–60P₂O₅ (10 ≤ x ≤ 40 mol%). The verification of successful glass compositions prior to heat treatment is already confirmed by observing unique glassy XRD halos observed in the as-prepared glass compositions. The addition of Cu_2_O is carried out with the intention of acting as a redox-active component and network modifier. The addition of Cu_2_O structurally disrupts the phosphate glass network by introducing non-bridging oxygen (NBO) sites, which is known to have a significant impact on defect structure and free volume^9,10^.

Compared to our earlier publication in Physica B^10^, which focused on the structural characterization, positron annihilation lifetime parameters, and electrical conductivity of the V₂O₅–Cu₂O–P₂O₅ glass–ceramic system, this study makes a notable scientific contribution. It establishes a direct link between free volume evolution and electrical transport. This work also presents a detailed analysis of vacancy size distribution using LT10 software (Version 10)^20,21^. It explains the difference between oxygen molar volume and PAL-derived localized free volume. Additionally, it investigates how nanocrystallization affects hopping conduction and proposes a detailed model that describes how free-volume defects influence electrical transport. Thus, this manuscript builds on the previous work by offering new insights into the mechanisms, rather than simply adding more experimental results^10^.

The novelty of the present work lies in combining X-ray diffraction, DC electrical conductivity measurements, and detailed positron annihilation lifetime spectroscopy—including free-volume size distribution analysis—to establish a direct structure defect transport relationship in xV₂O₅–(40 − x)Cu₂O–60P₂O₅ glass–ceramic nanocomposites (x = 10–40 mol%). In contrast to previous studies that treated structural and electrical properties separately, this work quantitatively demonstrates how vanadium incorporation and controlled heat treatment suppress vacancy-type defects, reduce free-volume size, and enhance small-polaron hopping conduction. Hence, the main aim of this research is to reveal the interplay between nanocrystallization, defect structures, and electrical transport in vanadium copper phosphate glass–ceramic nanocomposites. This microscopic understanding of the interplay will give further insights into defect engineering, which can be used to enhance transport properties in glass–ceramic materials based on transition metal oxides.

## Experimental set-up and data analysis

As starting ingredients, reagent-grade Cu_2_O (99.9%), V₂O₅ (99.9%), and P₂O₅ (99.99%) were utilized. All chemicals were purchased from Sigma Aldrich. The proper molar ratios were carefully mixed under ambient conditions to create a batch of 10 g. An electric furnace was then used to melt the mixture in a platinum crucible at 1250 K for an hour. After melting, the glass was quickly placed onto a sizable block of copper and instantly cooled by sandwiching it between two copper plates that were exactly alike. The bulk glass samples produced by this quenching method had a thickness of about 1 mm and dimensions of around 1 × 1 cm^2^. The samples were crystallized and then annealed in air for two hours at 873 K, which is close to the crystallization temperature, to create glass–ceramics nanocomposites.

X-ray diffraction (XRD) with a JEOL machine (JDX-9C, JEOL LTD) and CuKα radiation (wavelength = 1.5406 Å) verified the materials’ crystalline nature. The XRD measurements were conducted at the Central Laboratory of Microanalysis and Nanotechnology, Minia University. Using toluene as the immersion solvent and the Archimedes principle, the density of the glass samples was determined. DC electrical conductivity (σ_DC_) was measured between 303 and 588 K. Current–voltage (I-V) measurements were used to confirm the electrical behavior after silver paste electrodes were placed to the glass samples’ polished surfaces. To prepare the positron source, a 7 µm thick Kapton® foil was coated with about 740,000 Bq of aqueous ^22^NaCl solution. To encapsulate the ^22^NaCl layer, a second sheet of identical Kapton® foil was applied with epoxy adhesive. For a whole day, the sealed assembly was left to be cured in the open air. The short-lived components in the PAL spectrum are mostly caused by 10% of the emitted positrons that are absorbed by Kapton® foil, which is frequently utilized in positron lifetime measurements. The positron source correction was two components with lifetimes of 382 and 1741 ps and their intensities 97.26 and 2.74%, respectively. PAL spectra were captured by means of the conventional fast–fast coincidence method. Because Kapton® forms very few long-lived positronium states, it is especially well suited for time resolution calibration^22^. In light of this, the system’s time resolution—240 ps—was ascertained using the Kapton® PAL spectrum. The National Metrology Institute of Japan (AIST) provided certified reference materials, synthetic fused silica (NMIJ CRM 5601-a) and polycarbonate (NMIJ CRM 5602-a)^23^. Ito et al.^24^ stated that the values of the lifetimes are 1.623 ± 0.026 and 2.103 ± 0.038 ns for synthetic fused silica and polycarbonate, respectively. For the present PAL system, the lifetimes for the synthetic fused silica and polycarbonate are 1.60 ± 0.04 and 2.13 ± 0.05 ns, respectively. The positron lifetime data for the standard samples, which were measured using the present PAL system, are almost the same within the error bars, verifying our PAL system and guaranteeing accuracy. The PAL method was used in circumstances that were ideal for polymers and insulators, especially those with positronium lifetimes of more than one nanosecond. Every measurement was conducted three times to ensure precision and reproducibility.

Experimental positron lifetime data, denoted as *y*(*t*), can be described by a convolution (denoted by the symbol *) of the PAL system’s resolution function *Res*(*t*) with a finite sum of exponential decay components^25^;

1 $$y(t)=Res(t)*\left[{N}_{i}{\sum}_{i=1}^{n}({I}_{i}/{\tau}_{i})exp(-t/{\tau}_{i})+B\right]$$

The positron lifetimes and their corresponding relative intensities (with ∑ *I*_i_ = 1) are denoted by ${\tau}_{i}$ and* I*_*i*_, respectively, in this expression. *B* stands for the continuous background contribution, while Ni is the normalization factor. Non-linear least squares fitting was used to obtain these parameters. The PALSfit3 program^25^ was used to do the study, taking the source correction into account with total intensity 10% for each PAL spectrum containing approximately 3.0 × 10^6^ counts. In this investigation, an attempt is made to decompose the spectra into four components, but it gives unreliable variance ratio. The analysis of the PAL spectra showed that the best variance ratio (χ^2^ < 1.1) and most acceptable standard deviations were obtained when each spectrum is decomposed into three-lifetime components (i = 1 − 3). The first lifetime (τ_1_, *I*_*1*_) is the derived (generalized) value related to *p*-Ps lifetime and lifetime of positrons annihilating from defect-free Bloch states. The second lifetime (τ, *I*_*2*_) has a longer lifetime ranging and it is associated with the free positron annihilation. The third lifetime component (τ_3_, *I*_*3*_) has the longest lifetime and it results from *o*-Ps pick-off annihilation in the vacancies within the samples.

Previous studies have indicated that the PAL spectrum can also be represented by a continuous distribution model^20,26^, which is more realistic in certain disordered systems. This model is expressed as:

2 $$y\left(t\right)=Res\left(t\right)*\left[{N}_{t}{\int}_{0}^{\infty }(I(\tau )/\tau )\mathit{exp}(-t/\tau )d\tau +B\right]$$

where

3 $${\int}_{0}^{\infty }I\left(\tau \right)d\tau =1$$

and *N*_*t*_ is the total count in the PAL spectrum. For this analysis, the LT10 software package^20,21,26–28^ was used. This approach allowed for the extraction of the positronium annihilation rate distribution, λ = 1/τ_3_, particularly for the ortho-positronium (*o*-Ps) component associated with free volume or vacancies. The distribution of annihilation rates λ for *o*-Ps was assumed to follow a log-normal distribution^29^:

4 $${d\alpha }_{3}\left(\uplambda \right)=-{\left(2\pi \right)}^{-0.5}{\sigma}_{3}^{-1}exp\left\{-\frac{{\left[Ln\left(\uplambda \right)-Ln\left({\uplambda}_{30}\right)\right]}^{2}}{{2\sigma }_{3}^{2}}\right\}{\uplambda }^{-1}d\uplambda$$

where σ_3_ denotes the spread of the distribution, and *Ln* signifies the natural logarithm. Consequently, the *o*-Ps lifetime distribution α_3_ (τ_3_), can be written as:

$${d\alpha }_{3}\left({\uptau}_{3}\right)=-{\left(2\pi \right)}^{-0.5}{\sigma}_{3}^{-1}exp\left\{-\frac{{\left[Ln\left(\uplambda \right)-Ln\left({\uplambda}_{30}\right)\right]}^{2}}{{2\sigma }_{3}^{2}}\right\}{\uplambda }^{-1}d\uplambda \frac{{d\uptau }_{3}}{d\uplambda }$$

5 $$=-{\left(2\pi \right)}^{-0.5}{\sigma}_{3}^{-1}exp\left\{-\frac{{\left[Ln\left(\uplambda \right)-Ln\left({\uplambda}_{30}\right)\right]}^{2}}{{2\sigma }_{3}^{2}}\right\}{\uplambda }^{-1}{d\uptau }_{3}$$

here *λ*_*30*_ is the peak annihilation rate of positronium in vacancies.

PAL spectra can be used to quantitatively estimate the size of free volume or vacancies using the positronium hole theory^29^. This method uses the following relation^30,31^ to get the average radius of the free-volume voids (*R*_*v*_) using the ortho-positronium (*o*-Ps) lifetime component, represented as τ₃:

6 $${\tau}_{3}=0.5{\left\{1-\frac{{R}_{v}}{{R}_{o}}+\frac{1}{2\pi }sin\langle \frac{2\pi {R}_{v}}{{R}_{o}}\rangle \right\}}^{-1} \left(ns\right)$$

The relationship between *R*_*o*_​and *R*_*v*_​ can be expressed as *R*_*o*_ = *R*_*v*_ + *ΔR* where *ΔR* = 0.1656 nm. This value represents the thickness of the uniform electron layer in which positron annihilation occurs^32^. The volume of the vacancies *V*_*v*_ in cubic nanometers (nm^3^) is determined using this framework.

7 $${V}_{v}=\frac{4}{3}\pi {R}_{v}^{3}.$$

So that the vacancy size distribution was computed using Eqs. (4–7) and is expressed as^33^;

8 $${d\alpha }_{3}\left({V}_{v}\right)=-{\left(2\pi \right)}^{-0.5}{\sigma}_{3}^{-1}exp\left\{-\frac{{\left[Ln\left(\uplambda \right)-Ln\left({\uplambda}_{30}\right)\right]}^{2}}{{2\sigma }_{3}^{2}}\right\}{\uplambda }^{-1}d\uplambda \frac{d{V}_{v}}{d\uplambda }$$

where

$$\frac{d{V}_{v}}{d\uplambda }=-\frac{4\pi {R}_{v}^{2}{\left({R}_{v}+{R}_{o}\right)}^{2}}{2{R}_{o}}{\left\{1-cos\langle \frac{2\pi {R}_{v}}{{{R}_{v}+R}_{o}}\rangle \right\}}^{-1}$$

as derived from Eqs. (6 and 7).

## Results and discussion

### X-ray diffraction analysis

**Fig. 1:**
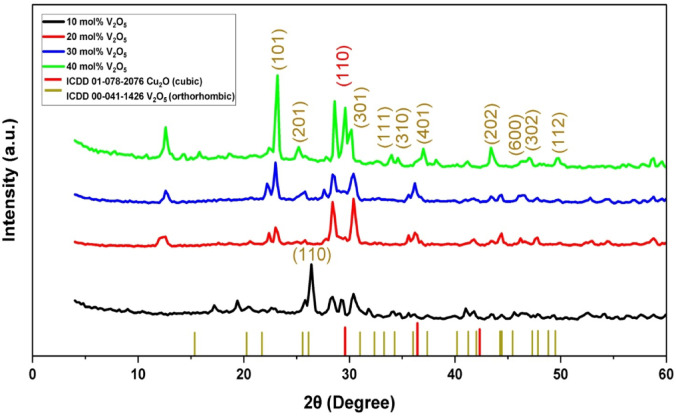
XRD patterns of xV₂O₅–(40 − x)Cu₂O–60P₂O₅ glass–ceramic nanocomposites (x = 10, 20, 30, and 40 mol%) after heat treatment at 873 K for 2 h. Vertical dashed lines represent standard diffraction peaks of Cu₂O (ICDD No. 01-078-2076) and V₂O₅ (ICDD No. 00-041-1426), confirming phase identification.

X-ray diffraction (XRD) is a widely used non-destructive technique for investigating the structural state of materials through the interaction of X-rays with atomic planes. It provides essential information regarding phase composition and degree of crystallinity. Figure 1 shows the XRD patterns of xV₂O₅–(40 − x)Cu₂O–60P₂O₅ glass–ceramic nanocomposites (x = 10–40 mol%) after heat treatment. The diffraction patterns reveal the coexistence of crystalline phases embedded within an amorphous matrix. At lower V₂O₅ content (10 mol%), the pattern is dominated by a broad background with relatively weak diffraction peaks, indicating a predominantly amorphous structure with limited crystallization.

As the V₂O₅ content increases (20–40 mol%), the intensity and sharpness of the diffraction peaks progressively increase, confirming the development of crystalline phases. The diffraction peaks observed around 2θ ≈ 29.4°, 36.2°, and 43.4° correspond to the (110), (111), and (200) planes of cubic Cu₂O (ICDD No. 01-078-2076)^34^. In addition, multiple peaks in the range 2θ ≈ 12–50° are indexed to orthorhombic V₂O₅ (ICDD No. 00-041-1426)^35^, including reflections such as (200), (101), (301), (310), and (401), as indicated in the figure. The presence of these peaks confirms the successful formation of the crystalline phases Cu₂O and V₂O₅ upon heat treatment. The increase in peak intensity and narrowing of peak width with increasing V₂O₅ content suggest enhanced crystallinity and the development of nanocrystalline domains. However, the persistence of the diffuse background indicates that the samples remain partially amorphous.

The average crystallite size (*D*) of the formed phases was estimated using the Scherrer equation:

10 $$D = K\lambda /\beta \cos \theta ,$$

where *K* is the shape factor (≈0.9), *λ* is the X-ray wavelength, *β* is the full width at half maximum (FWHM), and *θ* is the Bragg angle. The calculated crystallite sizes lie in the range of ~ 16–27 nm, confirming the formation of nanocrystalline phases. The ICDD reference lines shown at the bottom of the figure align well with the experimental peaks, supporting accurate phase identification. Overall, the results demonstrate that increasing V₂O₅ content promotes crystallization and phase development within the glass matrix. These structural changes are expected to influence charge transport by modifying the distribution of conduction pathways. High-resolution TEM characterization was not performed in the present study due to the current unavailability of the required facilities. TEM provides direct evidence of the crystallite size, morphology, and particle distribution, serving as an essential complement to XRD-based crystallite size estimation. These measurements will be carried out in future work.

The sizes of the crystallites were estimated using the strongest isolated diffraction peaks from the (111) reflection of Cu₂O and the (301) reflection of orthorhombic V₂O₅. Since no standard reference material was measured under the same instrumental conditions, we could not apply an instrumental broadening correction. Therefore, the calculated crystallite sizes should be seen as approximate values. Peak broadening may also result from microstrain and overlapping with the amorphous background. The estimated uncertainty in crystallite size is about ± 2 nm.

### DC electrical conductivity

**Fig. 2:**
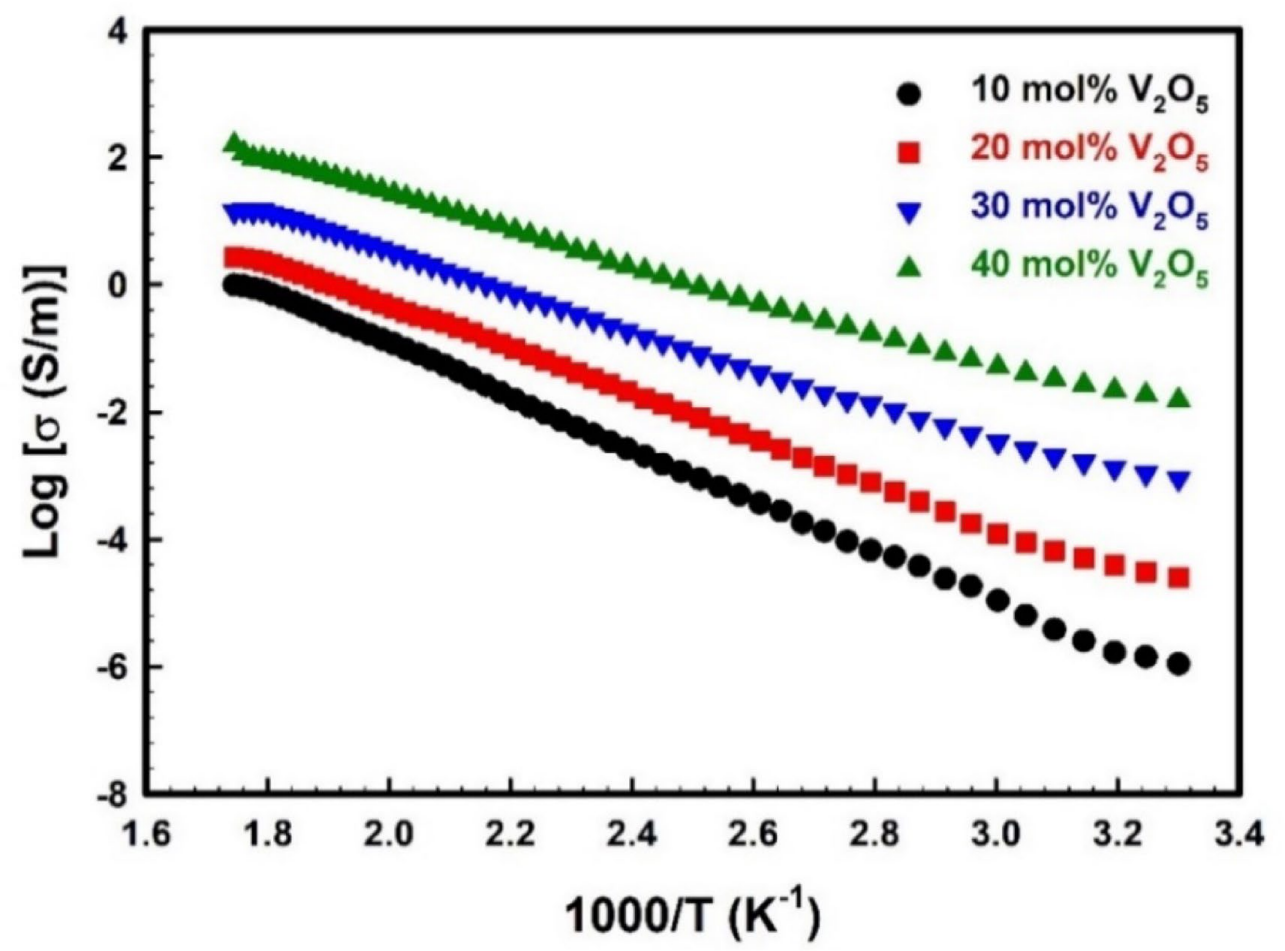
Temperature dependence of DC conductivity σ_DC_ as a function of reciprocal temperature 1/T for V_2_O_5_–Cu_2_O–P_2_O_5_ GCN.

Figure 2 presents the temperature dependence of DC electrical conductivity (σ_DC_) as a function of reciprocal temperature (1/*T*) for xV₂O₅–(40 − x) Cu₂O–60P₂O₅ GCN samples. The conductivity follows an Arrhenius-type behavior, described by Mott’s equation^36^, indicating thermally activated charge transport. The approximately linear relationship between log σ_DC_ and 1/*T* suggests that charge transport is dominated by hopping mechanisms typical of transition-metal oxide-containing glass systems. Deviations from linearity at higher temperatures may reflect temperature-dependent activation energy, consistent with previous studies^37^.

**Fig. 3:**
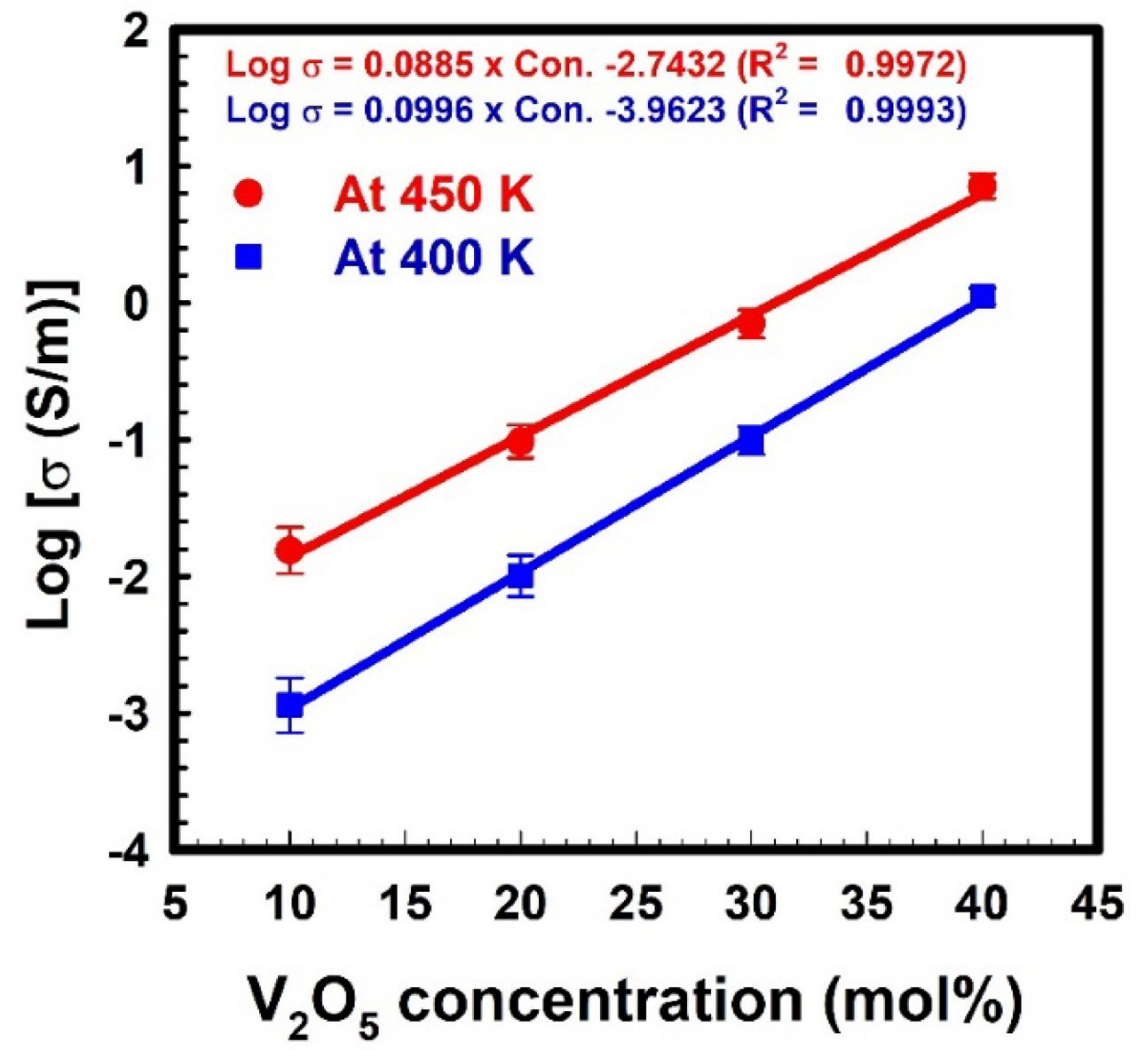
Effect of V_2_O_5_ content on DC conductivity at T = 400 and 450 K for V_2_O_5_–Cu_2_O–P_2_O_5_ GCN.

A systematic increase in σ_DC_ with increasing V₂O₅ concentration was observed. Instead of specifying the valence states, the increase in conductivity is ascribed to the overall contribution of the vanadium-related electronic states to the hopping conduction. The addition of V₂O₅ also causes changes to the phosphate network, including the generation of non-bridging oxygens (NBOs), which contribute to the mobility of the charge carriers and the increase in the density of the hopping states^38^. The conductivity increase for the heat-treated glasses is four orders of magnitude higher than that of the as-prepared glasses^10^. The increase in conductivity is primarily attributed to compositional effects, particularly the increased vanadium ion concentration and reduced interionic distance, while nanocrystalline phase formation provides a secondary structural contribution. As shown in Fig. 3, the values of the electrical conductivity at constant temperatures (400 and 450 K) increase with increasing vanadium concentration. This trend underlines the importance of the role played by vanadium oxide (V₂O₅) as a charge transporter. The combined addition of V₂O₅ and Cu_2_O considerably changes the glass structure, and the interaction with the phosphate network is important for the observed electrical properties.

The conductivity values obtained are similar to those reported for transition-metal phosphate glass ceramics. However, they are lower than those of highly crystalline V₂O₅ because the partially amorphous matrix leads to more carrier localization. Despite this, the observed four-order-of-magnitude increase in conductivity shows that nanocrystallization combined with higher vanadium concentration is effective.

### Activation energy and structural effects

**Fig. 4:**
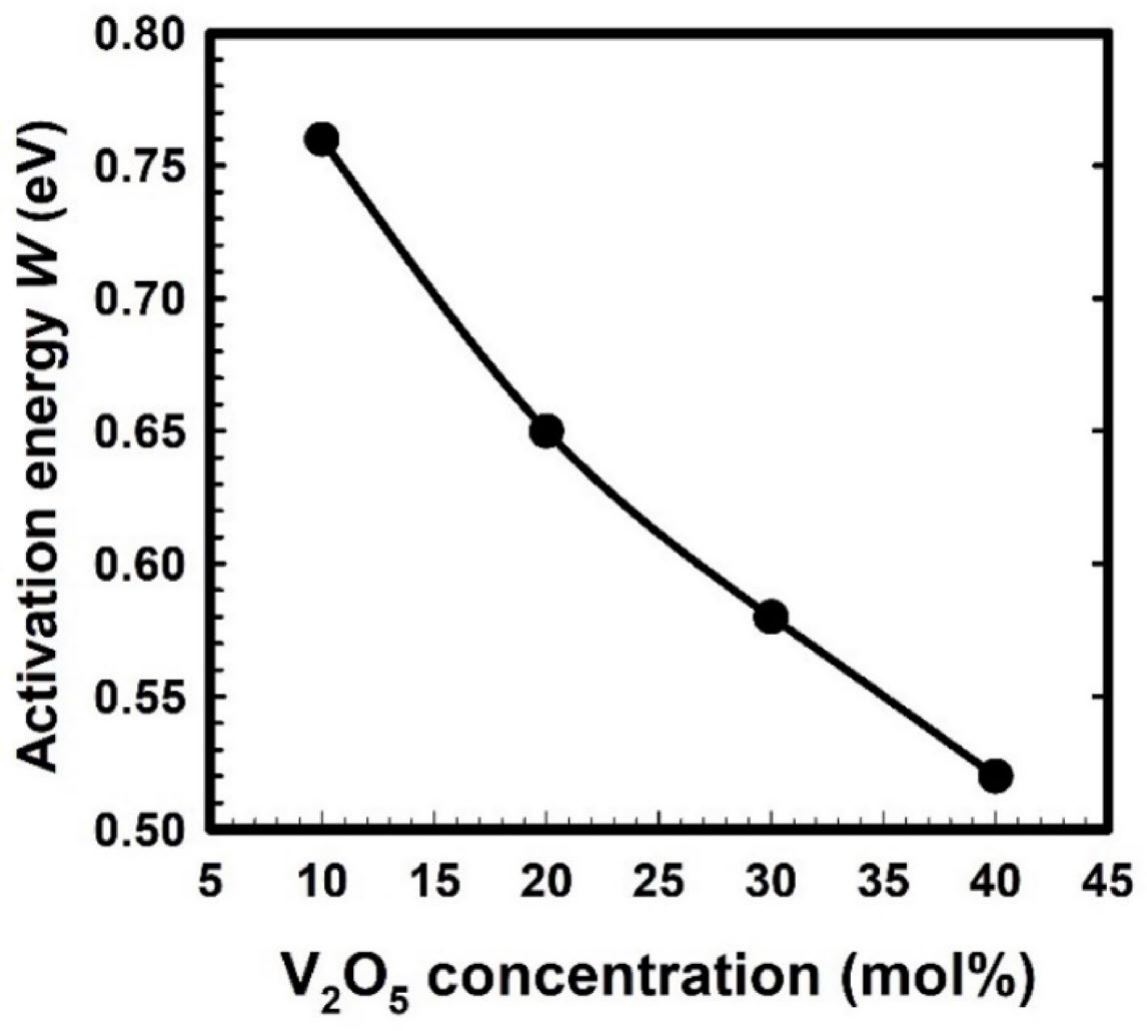
Effect of V_2_O_5_ content on activation energy *W* for V_2_O_5_-Cu_2_O–P_2_O_5_ GCN. The line is drawn to guide the eye. The errors are within the size of the symbol.

The activation energy for DC conduction is observed to decrease systematically with increasing V₂O₅ concentration [Fig. 4], signifying less resistance for charge carriers. This is related to increasing electronic state density and structural relaxation effects due to vanadium incorporation and heat treatment. As V₂O₅ is known for its network-modifying and electronic transport facilitation properties, its increasing concentration is observed to increase connectivity between localized states, as is typical for small polaron hopping mechanisms of transition metal phosphate glasses^39,40^.

### Density and oxygen molar volume

**Fig. 5:**
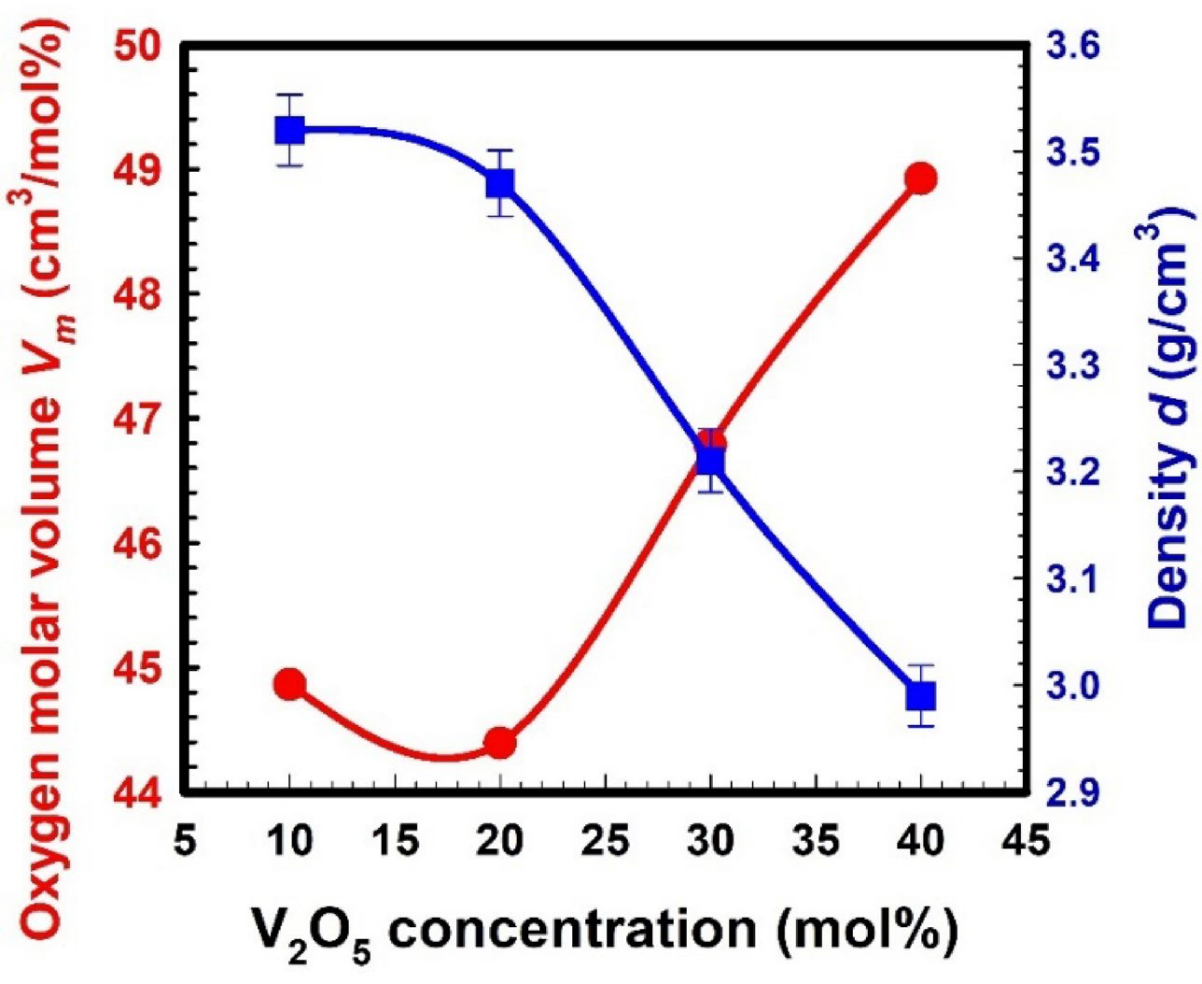
Composition dependence of density *d* and oxygen molar volume *V*_*m*_ for V_2_O_5_–Cu_2_O–P_2_O_5_ GCN. The lines are drawn to guide the eye.

Figure 5 shows the variation of density (*d*) and oxygen molar volume (*V*_m_) as a function of V₂O₅ concentration for the xV₂O₅–(40–x)Cu₂O–60P₂O₅ glass–ceramic nanocomposites. The results indicate a gradual decrease in density with increasing V₂O₅ content. This behaviour can be attributed to structural modifications occurring within the phosphate glass network. As V₂O₅ replaces Cu₂O in the composition, the glass structure becomes less densely packed due to changes in the coordination environment of the network-forming units. These structural rearrangements lead to an expansion of the glass network and a corresponding reduction in density. At the same time, the oxygen molar volume (*V*_m_) shows a systematic increase with increasing V₂O₅ concentration. The increase in* V*_m_ suggests a decrease in the packing efficiency of the glass network and indicates the presence of larger interstitial spaces or free-volume regions within the structure. This behaviour can be explained in terms of the polarizing power of the constituent cations, which is defined as the ratio of ionic charge to ionic radius. Vanadium cations possess a higher polarizing power compared with copper cations, resulting in stronger interactions with surrounding oxygen ions. This interaction modifies the local structural arrangement and causes a redistribution of oxygen ions within the glass network. The incorporation of vanadium oxide also leads to the formation of different structural units, such as VO₄ and VO₅ polyhedra, which influence the connectivity of the phosphate network. These structural units can distort the local bonding environment and contribute to an expansion of the glass structure, thereby increasing the oxygen molar volume.

Overall, the observed decrease in density and increase in oxygen molar volume with increasing V₂O₅ concentration indicate a progressive modification of the glass network structure. These changes are associated with variations in cation–oxygen interactions, ionic size effects, and the redistribution of structural units within the V₂O₅–Cu₂O–P₂O₅ glass–ceramic system, consistent with similar observations reported in related phosphate glass systems^41^.

### Electrical transport mechanism

In vanadium-containing phosphate glass–ceramic nanocomposites, DC electrical conductivity is predominantly governed by small-polaron hopping between mixed-valence vanadium ions (V^4^⁺/V^5^⁺). This conduction mechanism is highly sensitive to the spatial distribution and separation between vanadium ions within the glass network. The electrical conduction does not occur exclusively through interconnected V₂O₅ nanocrystals. Instead, electron transport proceeds through small-polaron hopping involving both vanadium ions remaining in the partially amorphous phosphate matrix and those located inside the nanocrystalline regions. Therefore, the nanocrystals act as highly conductive local domains, whereas the surrounding amorphous matrix still contributes to the overall hopping network. Cu₂O mainly acts as a network modifier by generating non-bridging oxygen sites and altering the local phosphate structure. Consequently, its contribution to electrical conductivity is primarily indirect through structural modification rather than direct electronic conduction. XRD confirms that the glass matrix remains partially amorphous after heat treatment. Consequently, charge transport results from the coexistence of crystalline V₂O₅ domains embedded within an electrically active amorphous matrix.

The average interionic distance (*A*_*id*_) between vanadium ions was calculated using the relation: *A*_*id*_ = (1/*N*)^1/3^, where *N* is the vanadium ion concentration per unit volume, determined from the experimentally measured density and glass composition. The calculated values of density (*d*), molecular weight (*M*_*w*_), vanadium ion concentration (*N*), and interionic distance (*A*_*id*_) are listed in Table 1. In addition, the polaron hopping distance *r*_*p*_ was estimated from the average interionic distance *A*_*id*_ using:

11 $${r}_{\mathrm{p}}={\left(\frac{\pi }{6}\right)}^{1/3}\frac{{A}_{id}}{2},$$

and also listed in Table 1. The results show that the hopping distance decreases with increasing V₂O₅ content. This reduction indicates that the separation between adjacent vanadium ions becomes smaller, thereby facilitating electron hopping and enhancing electrical conductivity. Furthermore, the values of the localization parameter *αA*_*id*_, which is used to represent the localization of charge carriers, are determined to be higher than unity, confirming the small polaron hopping conduction mechanism.

**Table 1:** Chemical composition and physical properties of V_2_O_5_–Cu_2_O–P_2_O_5_ GCN.

Nominal composition (mol%)			*d* ± 0.02 (g/cm^3^)	*W* ± 0.01 (eV)	*M*_*w*_ *(g/mol)*	*N* ± 0.01 (× 10^21^ cm^-3^)	*A*_*id*_ ± 0.01 (nm)	*r*_p_ ± 0.01 (nm)	*N*(*E*_F_) (× 10^21^ eV^-1^cm^-3^)
V_2_O_5_	Cu_2_O	P_2_O_5_							
10	30	60	3.52	0.76	157.917	2.33	0.7542	0.3039	0.73
20	20	60	3.47	0.65	154.037	4.60	0.6015	0.2424	1.69
30	10	60	3.21	0.58	150.167	6.38	0.5393	0.2173	2.63
40	0	60	2.99	0.52	146.285	7.92	0.5017	0.2021	3.64

It should be emphasized that A_id_ and r_p_ represent effective bulk-average parameters derived from the overall vanadium concentration. Because the present material consists of V₂O₅ nanocrystals dispersed within a partially amorphous matrix, these parameters do not represent the exact V–V distances inside individual nanocrystals. They should therefore be interpreted as average transport descriptors useful for discussing composition-dependent conductivity trends.

The density of localized states near the Fermi level, *N(E*_*F*_*)*, was estimated using the expression proposed by Emin and Holstein^42^:

12 $$\it N\left({E}_{f}\right)=\frac{3}{4 \uppi {Aid}^{3 }\mathrm{W}}$$

The calculated values of *N(E*_*F*_*)* are also listed in Table 1. The obtained values fall within the typical range reported for disordered transition metal oxide glasses and are characteristic of localized electronic states associated with polaronic conduction. Moreover, the gradual increase in *N(E*_*F*_*)* with increasing V_2_O_5_ concentration indicates a higher density of localized states available for electron hopping, which further contributes to the enhancement of electrical conductivity in the investigated glass ceramic nanocomposites.

**Fig. 6:**
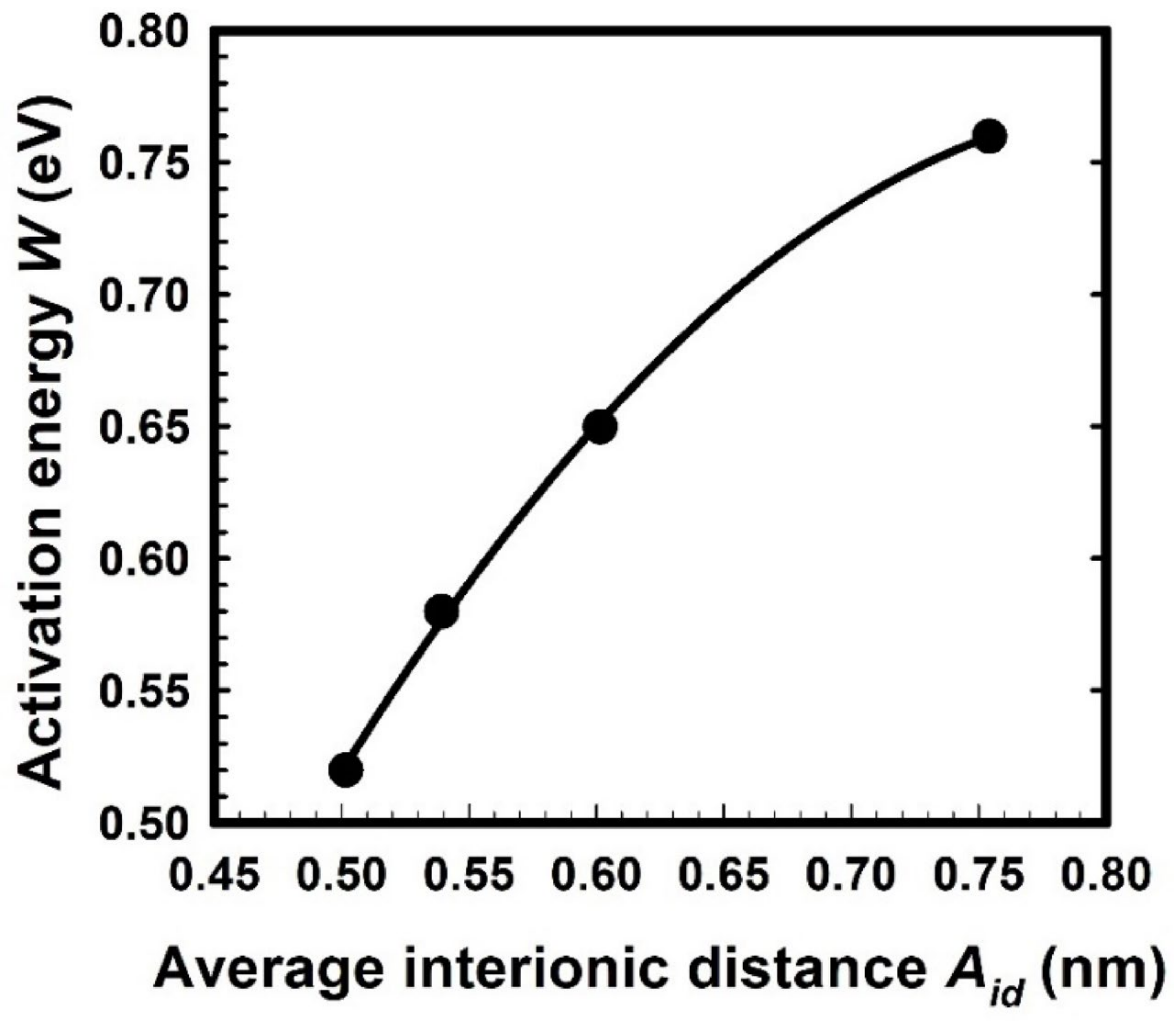
Variation of average interionic distance *A*_*id,*_ and the high-temperature activation energy, *W,* for V_2_O_5_–Cu_2_O–P_2_O_5_ GCN. The line is drawn to guide the eye.

As the concentration of V₂O₅ is increased from 10 to 40 mol%, the concentration of vanadium ions (*N*) also increases significantly, resulting in a corresponding decrease in interionic distance (*A*_*id*_) and polaron hopping distance (*r*_*p*_). This helps in the overlapping of orbitals between the vanadium ions, resulting in increased electron hopping and hence the conductivity. Simultaneously, the activation energy (*W*), obtained from Arrhenius analysis (Section "Activation energy and structural effects"), decreases with decreasing interionic distance, as shown in Fig. 6. This inverse relationship indicates that shorter hopping distances reduce the energy barrier for charge transport. These results clearly demonstrate that the electrical conduction in the present glass–ceramic nanocomposites occurs via a thermally activated small-polaron hopping mechanism, and that increasing V₂O₅ content enhances electrical transport by reducing hopping distance and activation energy. These findings are consistent with previous reports by Sayer and Mansingh^43^ and El-Desoky^44^, who demonstrated that increased V–O–V separation leads to higher hopping activation energies. On the other hand, the increase in conductivity is mainly attributed to the reduction in interionic distance between vanadium ions (V^4^⁺/V^5^⁺ pairs). As the V₂O₅ content increases, the vanadium ion concentration rises, leading to a decrease in both the average interionic distance (A_id_) and polaron hopping distance (r_p_). This enhances the probability of small-polaron hopping and reduces the activation energy for conduction.

### PAL analysis

In the present study, the term ‘defects’ refers primarily to free-volume (vacancy-type) sites within the glass network, as probed by PAL spectroscopy. These defects correspond to open-volume cavities associated with local structural disorder rather than crystallographic defects. The PAL spectra of the xV₂O₅–(40–x)Cu₂O–60P₂O₅ glass–ceramic nanocomposites (GCN), where x = 10, 20, 30, and 40 mol%, were analysed by decomposing the spectra into three lifetime components (τ₁, τ₂, and τ₃) using the PALSfit and LT10 analysis programs. The PAL parameters obtained from both fitting procedures showed close agreement, confirming the reliability of the lifetime analysis. In disordered systems such as oxide glasses and glass–ceramic nanocomposites, three lifetime components are typically observed. The shortest lifetime component (τ₁) corresponds to the annihilation of free positrons in defect-free regions of the matrix or shallow trapping sites. The intermediate lifetime component (τ₂) is generally associated with positrons trapped at vacancy-type defects or structural imperfections within the amorphous network. The longest lifetime component (τ₃) is attributed to the annihilation of ortho-positronium (*o*-Ps) formed in free-volume cavities or nano-voids in the glass structure^45,46^.

**Fig. 7:**
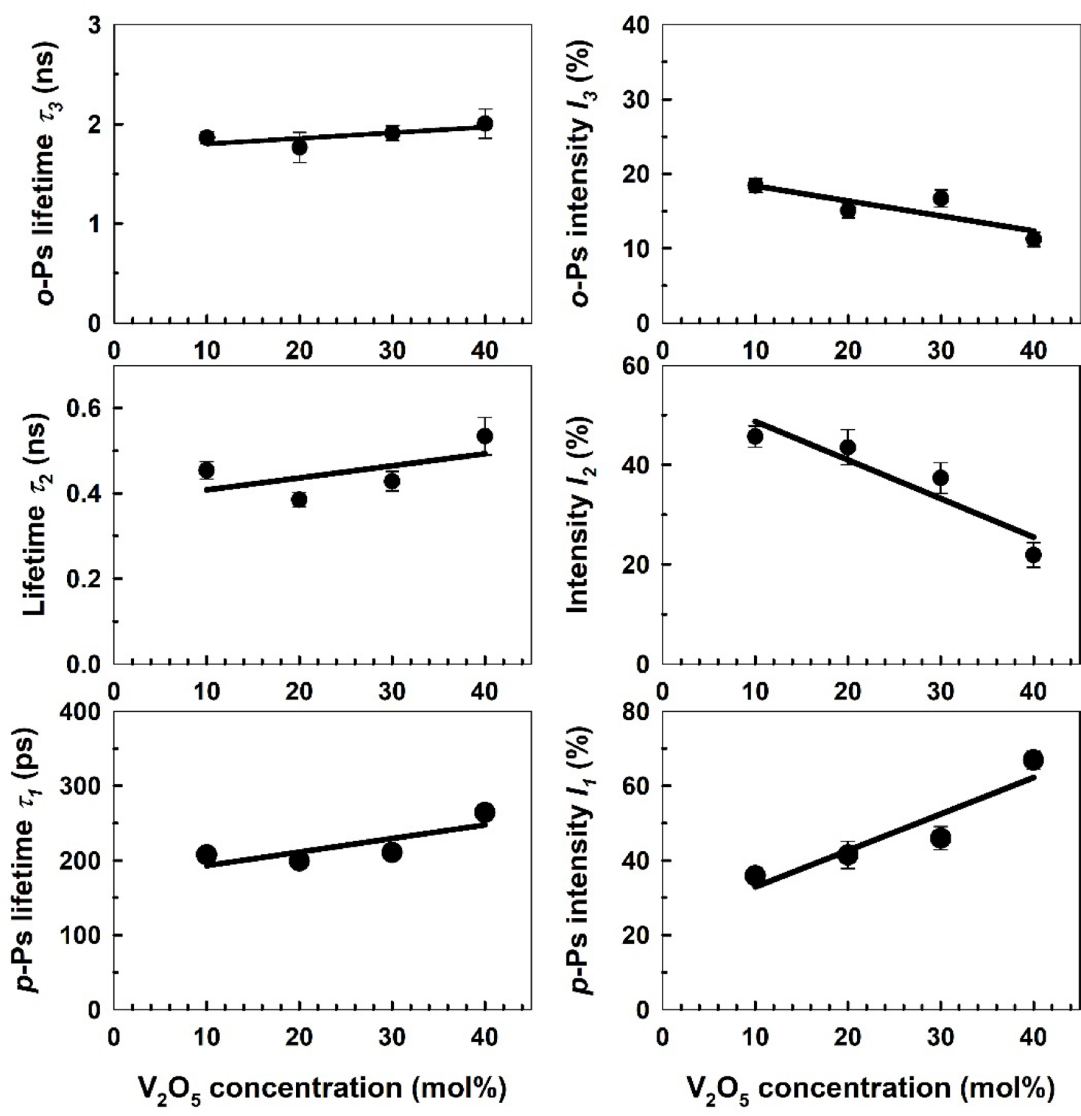
The three lifetimes’ components (τ_1_, τ_2_, and τ_3_) and their intensities (*I*_*1*_, *I*_2_, and *I*_3_) for *x*V_2_O_5_–(40−*x*)Cu_2_O–60P_2_O_5_ GCN with different concentrations of V_2_O_5_. The lines are drawn to guide the eye.

The corresponding intensities (*I₁, I₂*, and I₃) represent the probabilities of positron annihilation through these channels. The variations of these parameters with V₂O₅ concentration are presented in Fig. 7. These variations reflect modifications in the defect structure and free-volume distribution of the glass–ceramic matrix caused by the incorporation of vanadium oxide. The intermediate lifetime component τ₂ was found to lie in the range 0.38–0.53 ns, which is characteristic of positron trapping at vacancy-type defects in oxide glasses. The presence of such defects may arise from structural disorder, non-stoichiometric bonding configurations, or local distortions within the phosphate glass network. The gradual change in τ₂ with increasing V₂O₅ concentration indicates that the incorporation of vanadium ions modifies the local defect environment of the glass matrix. The longest lifetime component τ₃ corresponds to the annihilation of ortho-positronium atoms in free-volume holes within the glass structure. The variation of τ₃ and its intensity *I₃* provides important information about the size and concentration of these free-volume cavities. The observed decrease of τ₃ with increasing V₂O₅ concentration suggests that the average size of free-volume holes decreases as vanadium oxide is incorporated into the glass network. This behaviour indicates a gradual compaction of the glass structure and a redistribution of free-volume sites.

**Fig. 8:**
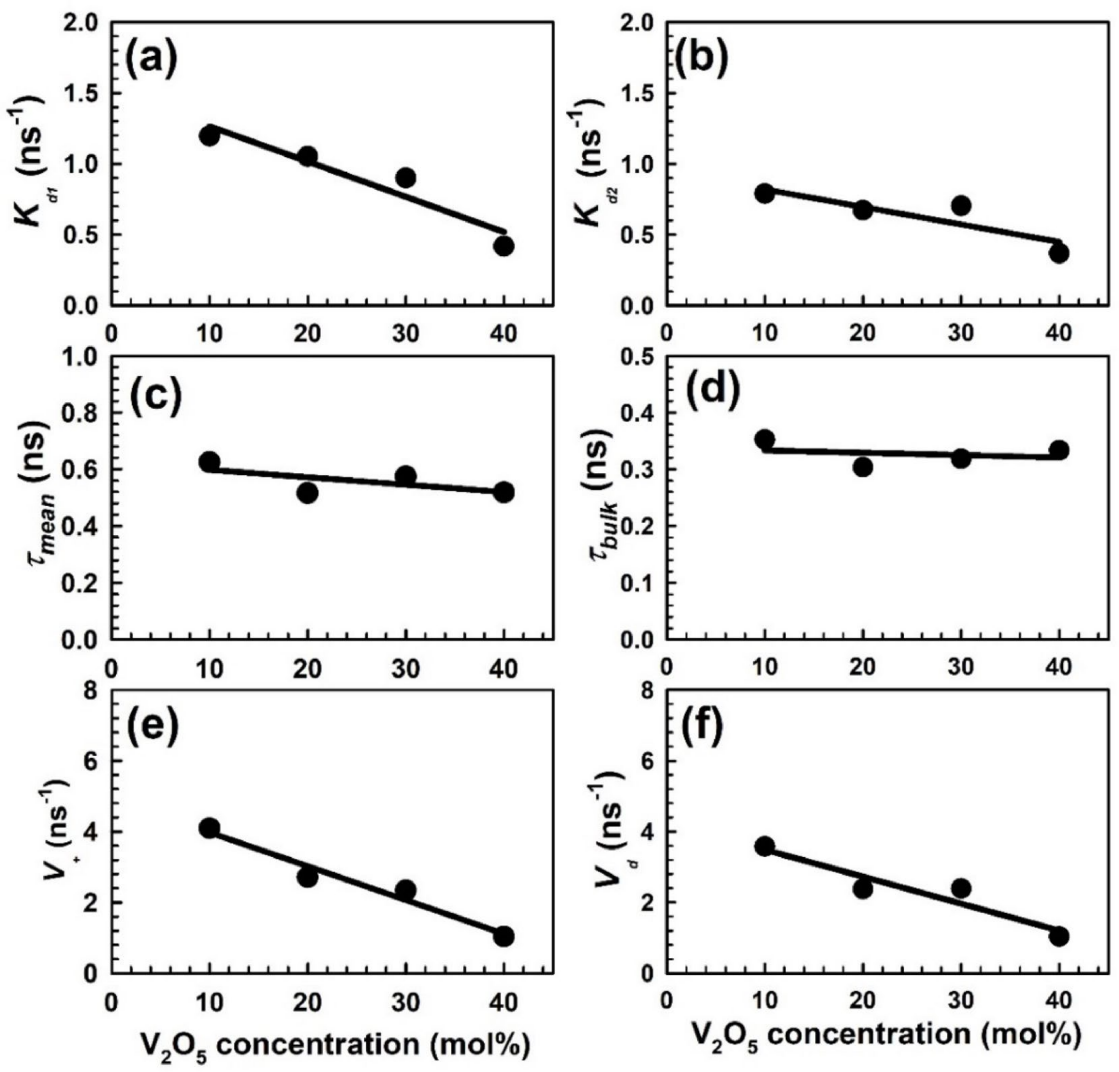
Variation of (**a**) *K*_*d1*_, (**b**)* K*_*d2*_, (**c**) τ_mean,_ (**d**) τ_bulk,_ (**e**) *V*_+_, and (**f**) *V*_*d*_ for *x*V_2_O_5_–(40-*x*)Cu_2_O–60P_2_O_5_ GCN with different concentrations of V_2_O_5_. The lines are drawn to guide the eye and the errors are within the size of the symbols.

According to the two-state positron trapping model proposed by Shpotyuk et al.^47^ and Šedivý et al.^48^, positrons may become trapped at different defect sites within the material. In this model, positron annihilation occurs either in defect-free regions or in trapping centres such as vacancies or free-volume holes. Although the two-state model does not explicitly distinguish between different types of defects associated with multiple lifetime components, it provides a useful framework for estimating the positron trapping rates. The trapping rates for positrons at different defect sites were calculated using the relations:

13 $${K}_{d1}={I}_{2}\left(\frac{1}{{\tau}_{1}}-\frac{1}{{\tau}_{2}}\right),$$

14 $${K}_{d2}={I}_{3}\left(\frac{1}{{\tau}_{1}}-\frac{1}{{\tau}_{3}}\right).$$

where *K*_*d1*_ represents the trapping rate associated with vacancy-type defects, and *K*_*d2*_ corresponds to trapping related to free-volume sites where positronium formation occurs. The variation of *K*_*d1*_ and *K*_*d2*_ as a function of V₂O₅ concentration is shown in Fig. 8a, b. The opposite trends observed for these parameters indicate that the addition of V₂O₅ significantly influences the defect structure of the glass–ceramic nanocomposites. As the vanadium content increases, the trapping rate associated with vacancy-type defects decreases, suggesting a reduction in the concentration of these defects within the glass network.

The mean positron lifetime (τ_mean_), which reflects the overall defect structure of the samples, was calculated using^49,50^:

15 $${\uptau}_{\mathrm{m}\mathrm{e}\mathrm{a}\mathrm{n}}=\frac{{\uptau}_{1}{I}_{1}+ {\uptau}_{2}{I}_{2}+ {\uptau}_{3}{I}_{3} }{{I}_{1}+{I}_{2}+{I}_{3} },$$

The variation of τ_mean_ with V₂O₅ concentration is shown in Fig. 8c. The results reveal a gradual decrease in τ_mean_ as the V₂O₅ concentration increases, indicating a reduction in the average defect size or free-volume cavities within the glass matrix. The bulk positron lifetime τ_bulk_ corresponding to annihilation in defect-free regions of the material was determined from the relation:

16 $${\uptau}_{\mathrm{b}\mathrm{u}\mathrm{l}\mathrm{k}}= {\left[\frac{{I}_{1}}{{\uptau}_{1}}+\frac{{I}_{2}}{{\uptau}_{2}}+\frac{{I}_{3}}{{\uptau}_{3}}\right]}^{-1}$$

The calculated τ_bulk_ values are presented in Fig. 8d. A gradual decrease in τ_bulk_ with increasing V₂O₅ concentration suggests an increase in the annihilation rate of free positrons within the matrix, which may be related to structural rearrangements in the glass network.

The trapping rate of positrons in vacancy-type defects (*V*_+_) was estimated using the expression proposed by Shantarovich and Goldanskii^51^:

17 $${V}_{+}=\frac{3{I}_{2}\left(\frac{1}{\langle {\tau}_{b}\rangle }-\frac{1}{{\tau}_{2}}\right)}{3{I}_{1}-{I}_{3}}.$$

The obtained values of *V*_+_ are shown in Fig. 8e. The results indicate that *V*_+_ decreases with increasing V₂O₅ concentration, suggesting a reduction in the density of vacancy-type defects in the glass matrix. Similarly, the positronium trapping rate (*V*_*d*_) associated with free-volume sites was calculated using^52^:

18 $${V}_{d}=\frac{4{I}_{3}\left(\frac{1}{\langle {\tau}_{b}\rangle }-\frac{1}{{\tau}_{3}}\right)}{3-4{I}_{3}-3{I}_{2}},$$

The calculated values of *V*_*d*_ are shown in Fig. 8f. The gradual decrease of *V*_*d*_ with increasing V₂O₅ concentration further supports the conclusion that the incorporation of vanadium oxide leads to a reduction in the size and concentration of free-volume cavities within the glass network.

**Fig. 9:**
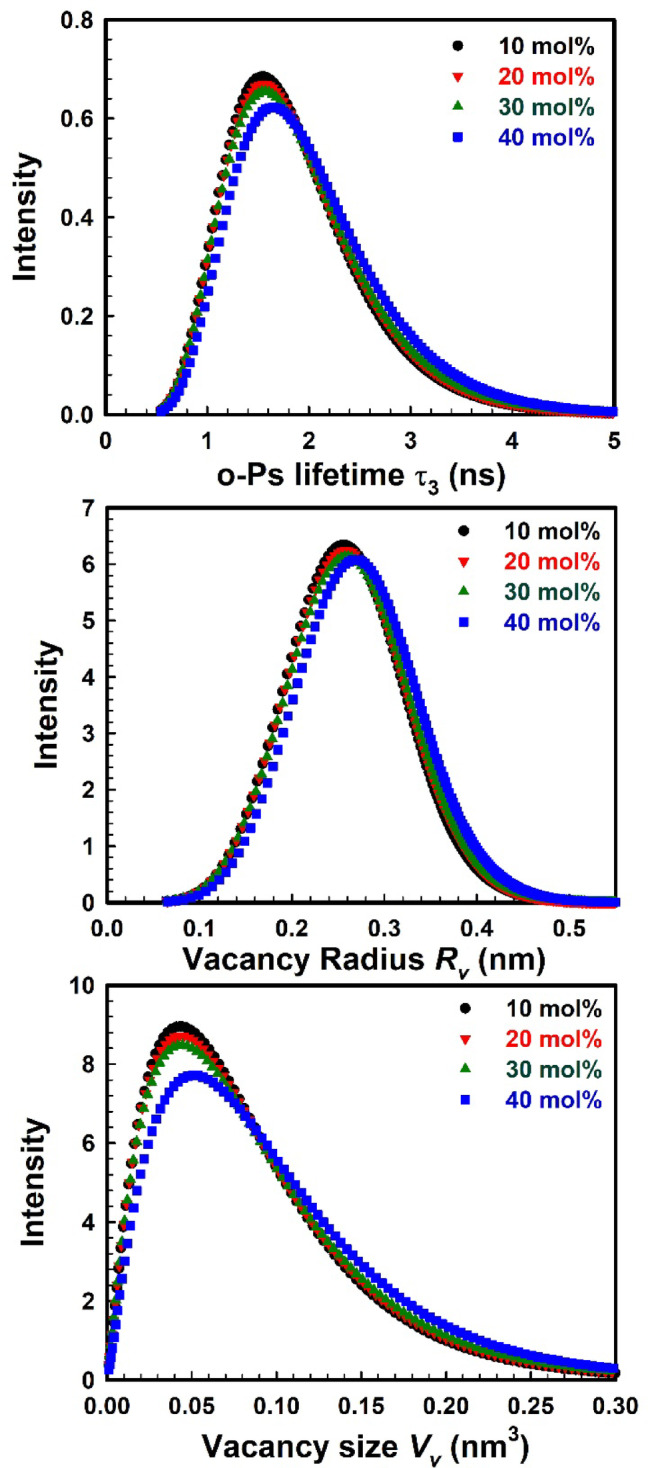
Distribution of long lifetime τ_3_, the vacancy radius *R*_*v*_, and vacancy size *V*_*v*_ for *x*V_2_O_5_–(40-*x*)Cu_2_O–60P_2_O_5_ GCN with different concentrations of V_2_O_5_ (where *x* = 10, 20, 30, and 40 mol%) deduced using LT10 program.

The structural parameters associated with the longest lifetime component τ₃, including the vacancy radius (*Rv*) and the free-volume size (*V*_*v*_), were estimated using the LT10 analysis program^25,53^. The distributions of these parameters are presented in Fig. 9. The results show that both the vacancy radius and the corresponding free-volume size decrease slightly with increasing V₂O₅ concentration, indicating a gradual compaction of the glass network structure. The calculated full width at half maximum (FWHM) values of the vacancy size distribution for V₂O₅ concentrations of 10, 20, 30 and 40 mol% were 0.1040, 0.1043, 0.1064, and 0.1174 nm^3^, respectively. These values suggest a slight narrowing of the free-volume distribution with increasing vanadium content.

Overall, the PAL results demonstrate that the addition of V₂O₅ significantly modifies the structural features of the xV₂O₅–(40–x)Cu₂O–60P₂O₅ glass–ceramic nanocomposites. The observed changes in positron lifetimes, trapping rates and free-volume parameters indicate a gradual reduction in vacancy-type defects and free-volume cavities as the V₂O₅ concentration increases, reflecting the important role of vanadium oxide in controlling the structural properties of the investigated glass system. However, the positron annihilation lifetime (PAL) results indicate a reduction in vacancy-type defects and free-volume size, which reflects improved structural ordering (or structural compactness). This structural refinement reduces disorder and carrier localization, thereby facilitating more efficient electron hopping. Although free-volume (vacancy-type) defects decrease with increasing V₂O₅ content, the enhancement in conductivity is dominated by compositional effects, while defect reduction contributes indirectly by reducing carrier localization. It should be noted that the increase in oxygen molar volume observed from density measurements does not contradict the reduction in PAL-derived free-volume size and vacancy-related parameters. The oxygen molar volume reflects a macroscopic structural parameter associated with the overall rearrangement and expansion of the phosphate glass network due to the incorporation of V₂O₅ and the formation of VO₄/VO₅ structural units. In contrast, PAL spectroscopy probes microscopic localized free-volume cavities and vacancy-type defects within the glass matrix. Therefore, although the average network structure becomes relatively more expanded at the macroscopic scale, the local structural ordering and homogenization improve simultaneously, leading to a reduction in the size and concentration of localized free-volume defects. These observations indicate that the structural evolution occurs at different length scales and collectively contributes to the observed electrical transport behavior.

### Correlation between electrical conductivity and defect structure

**Fig. 10:**
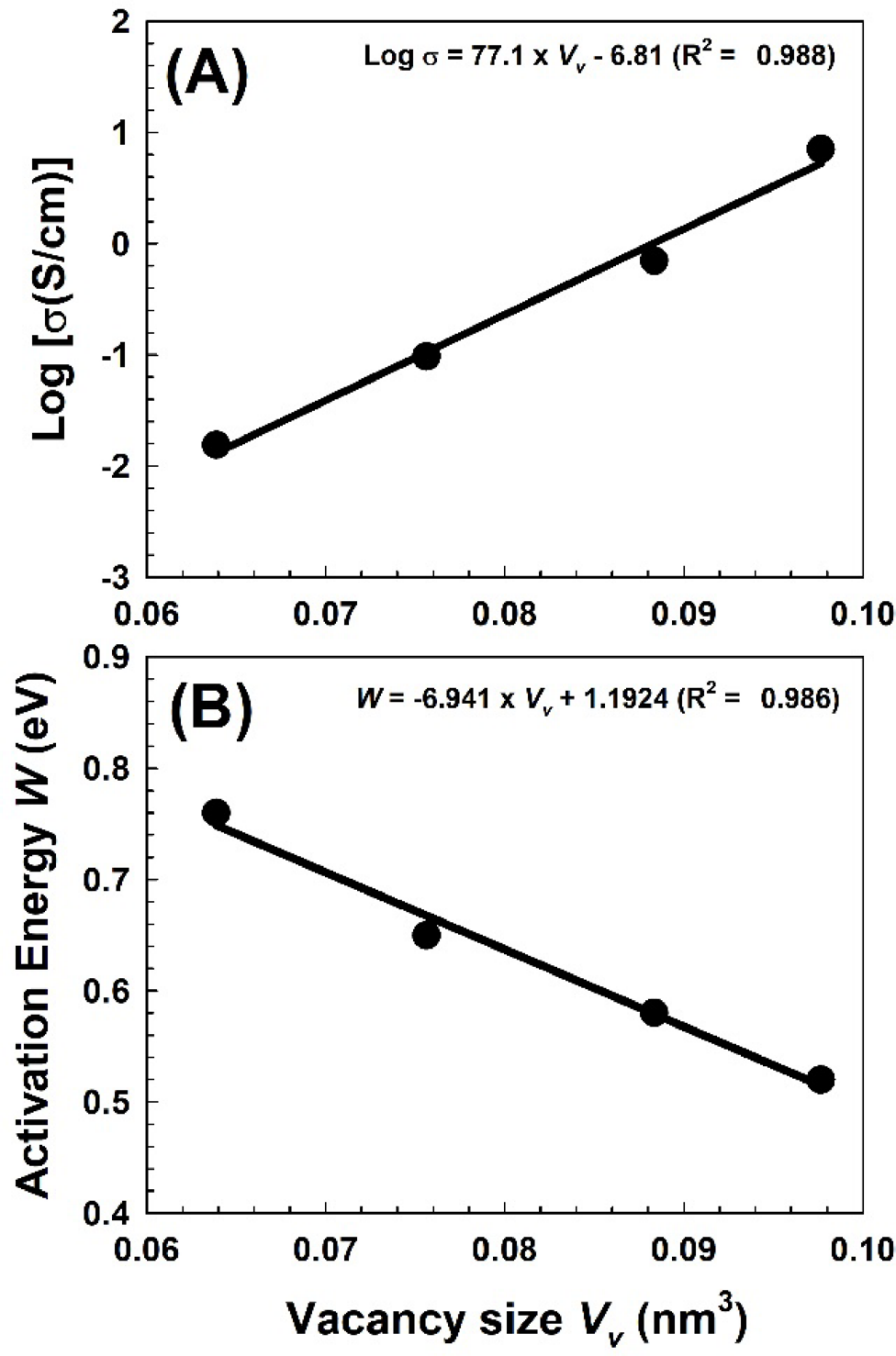
(**A**) Log(σ_DC_) and (**B**) the activation energy at high temperature for xV_2_O_5_–(40-x)Cu_2_O–60P_2_O_5_ GCN with varying V_2_O_5_ concentrations (where x = 10, 20, 30, and 40 mol%) are correlated with the vacancy size *V*_*v*_. The errors are within the size of the symbols.

Figure 10 illustrates the relationship between DC electrical conductivity (σ_DC_), activation energy (*W*), and vacancy size (*V*_*v*_) obtained from PAL analysis for xV₂O₅–(40–x)Cu₂O–60P₂O₅ glass–ceramic nanocomposites. As shown in Fig. 10A, the logarithm of DC conductivity increases with increasing vacancy size. However, although the vacancy size decreases with increasing V₂O₅ content, the conductivity enhancement is primarily governed by compositional effects, including the increased concentration of vanadium ions and the reduction in interionic and hopping distances. In this context, defect-related changes influence electrical transport indirectly by modifying the local structural environment. Figure 10B shows that the activation energy decreases with increasing vacancy size, indicating that free-volume (vacancy-type) defects can reduce the energy barrier for charge transport by facilitating thermally activated hopping. These defects correspond to free-volume cavities probed by PAL and are distinct from the crystalline features identified by XRD. Their role is mainly indirect, as they modify local atomic packing and orbital overlap between neighbouring transition-metal ions.

Overall, the results indicate that electrical transport is dominated by small-polaron hopping between V^4^⁺/V^5^⁺ ions, controlled primarily by compositional parameters such as vanadium ion concentration and interionic distance. The reduction in free-volume defects with increasing V₂O₅ content reflects improved structural ordering, which supports charge transport by reducing carrier localization. Thus, defect evolution, network modification, and compositional effects collectively influence the transport behaviour, with composition playing the dominant role.

## Conclusions

The xV₂O₅–(40 − x)Cu₂O–60P₂O₅ glass–ceramic nanocomposites exhibit a clear relationship between structural modification, free-volume characteristics, and electrical transport behavior. XRD analysis confirmed the formation of Cu₂O and V₂O₅ nanocrystalline phases with crystallite sizes in the range of ~ 16–27 nm after heat treatment. DC electrical conductivity increased significantly with increasing V₂O₅ concentration, while the activation energy decreased, indicating enhanced thermally activated small-polaron hopping conduction. The conductivity enhancement is mainly attributed to compositional effects, particularly the increase in vanadium ion concentration and the reduction in interionic and hopping distances between neighboring vanadium ions. PAL analysis revealed a gradual decrease in vacancy-type defect size and free-volume parameters with increasing V₂O₅ content, suggesting improved local structural ordering and reduced carrier localization. Although the oxygen molar volume increased slightly, this behavior reflects macroscopic structural rearrangement of the glass network rather than an increase in localized free-volume cavities. Thus, the increase in oxygen molar volume and the reduction in PAL-derived free volume occur at different structural scales and are not contradictory. Overall, the results demonstrate that electrical transport in the investigated glass–ceramic nanocomposites is predominantly governed by composition-dependent hopping parameters, while defect evolution contributes indirectly through modifications in structural compactness and local ordering. These findings provide additional insight into the interplay between structure, free-volume defects, and charge transport in transition-metal oxide glass–ceramic systems.

## Data Availability

The datasets used and/or analysed during the current study available from the corresponding author on reasonable request.
